# Word’s Contextual Predictability and Its Character Frequency Effects in Chinese Reading: Evidence From Eye Movements

**DOI:** 10.3389/fpsyg.2020.01833

**Published:** 2020-08-07

**Authors:** Zhifang Liu, Xuanwen Liu, Wen Tong, Fuyin Fu

**Affiliations:** ^1^Department of Psychology, College of Education, Hangzhou Normal University, Hangzhou, China; ^2^Department of Psychology, College of Education, Shaanxi Normal University, Linfen, China; ^3^Department of Psychology, College of Education, Ningbo University, Ningbo, China

**Keywords:** Chinese reading, word predictability, characters frequency, eye movements, word segmentation

## Abstract

The present study sought to establish how a word’s contextual predictability impacts the early stages of word processing when reading Chinese. Two eye-movement experiments were conducted in which the predictability of the target two-character word was manipulated; the frequency of the target’s initial character was manipulated in Experiment 1, as was the target’s end character frequency in Experiment 2. No reliable interaction effect of predictability with initial character frequency was observed in Experiment 1. Reliable interactions of word predictability with end character frequency were observed in Experiment 2. The end character frequency effects, in which the words with high-frequency end characters were fixated for a shorter time and re-fixated less often, were only observed when reading unpredictable words. Reliable interactions were also observed with incoming saccade length, as high-frequency end character words elicited longer saccades to themselves than low-frequency end character words when reading predictable words. The effects of pervasive predictability on measures of fixation time, probability, and saccade length were noted in both experiments. Our findings suggest that a word’s contextual predictability facilitates the processing of its constituent characters.

## Introduction

It has been extensively documented that the contextual predictability of words in a given context is closely related to how easily they can be processed during reading. In eye movement research, so-called predictability effects are exemplified by the fact that readers fixate on words that are predictable from the preceding context more quickly than words that are unpredictable; furthermore, predictable words are skipped more frequently than unpredictable words. These effects are robust and have been demonstrated in alphabetic languages, such as English and French (Ehrlich and Rayner, 1981; Balota et al., 1985; Fischler, 1985; Schustack et al., 1987; Altarriba et al., 1996; Rayner and Well, 1996; Rayner et al., 2001; Ashby et al., 2005; Bélanger and Rayner, 2013). The temporal resolution of the event-related potentials (ERPs) technique has also been used to determine how context affects word recognition. A well-replicated finding using this technique is that N400 amplitudes are inversely proportional to the contextual predictability, with a low-predictability word eliciting a more negative N400 than a high- predictability word (Dambacher et al., 2006; Dambacher and Kliegl, 2007).

The most common approach to gauge the temporal course of contextual predictability effects has been to observe the interaction of predictability with word frequency (Sternberg, 1969). The presence of word frequency effects is considered a marker for lexical access from bottom-up processing (Hudson and Bergman, 1985; Monsell et al., 1989; Sereno and Rayner, 2000, 2003). In general, word recognition can be subdivided into three stages: pre-lexical, lexical, and post-lexical processing. Pre-lexical processing of visual words includes process-related components such as visual analysis, word-form perception, and extraction of orthographic, phonological, and semantic features (Forster, 1981; Fodor, 1983). The “modular” view proposes that word processing in sentences can be initiated only after the physical properties of the stimulus are received and context can only exert its effect at the post-lexical stage for semantic integration. Thus, the modular view does not predict an interaction between word predictability and frequency factors on word processing in the lexical processing stage (i.e., lexical access) or in the early pre-lexical processing stages previously mentioned. An alternative view on how context affects the bottom-up stream of word recognition, called the “interactive account,” predicts an immediate mutual influence at various levels of lexical processing (Morton, 1969; McClelland and Rumelhart, 1981) so that contextual information can exert its effect from the early stages of word recognition, such as the early perceptual features analysis, to the later stage of lexical activation and selection (Federmeier, 2007).

Ample evidence has shown no reliable statistical interaction between contextual predictability and word frequency on eye movement measures during silent reading of alphabetic languages (Rayner et al., 2004, 2006; Ashby et al., 2005; Miellet et al., 2007; Lu et al., 2008; Hand et al., 2010; Gollan et al., 2011; Slattery et al., 2012). Evidence has demonstrated that predictability can facilitate the preprocessing of a word being viewed parafoveally through the extraction of its visual, orthographic, phonological, and semantic features in alphabetic language reading (Balota et al., 1985; White et al., 2005; Schotter et al., 2012, 2015). ERP components have been used to index various stages of lexical processing and the evidence suggests that the impact of contextual information on the ERP components starts very early and stretches into later time windows (Federmeier and Kutas, 2001; Penolazzi et al., 2007). Additionally, Sereno et al. (2003) demonstrated that context interacts with word frequency on the N1 component, 132–192 ms after word onset. The N1 component is always considered to be an index of visual processing. Thus, the finding of Sereno et al. (2003) suggests that context has an impact from the early stages of alphabetic word processing.

Chinese text is printed as a sequence of equally spaced, box-like characters, with most words consisting of two or more characters. As a logographic writing system, Chinese text is drastically different from the alphabetic text in how meaning is represented. Despite the great differences between Chinese and alphabetic scripts, evidence has shown similar contextual predictability effects, and also similar additive effects of contextual predictability and word frequency on eye movement measures in simplified Chinese reading (Rayner et al., 2005; Lu et al., 2008). Liu et al. (2018) observed predictability effects on saccade length, with high-predictability words eliciting longer saccades to themselves than low-predictability target words, suggesting that predictability facilitates parafoveal processing in Chinese reading. A study with the ERPs technique conducted by Lee et al. (2012) also observed a reliable interaction of predictability and word frequency at the anterior N1 component in traditional Chinese word-by-word reading. In their study, it was found that the predictability effect, in which a low-predictability word elicited a more negative N1 than a high-predictability word, was only obtained when reading high-frequency words, thus also suggesting that context facilitates early word processing stages when reading traditional Chinese.

It should be noted that the rapid serial visual presentation (RSVP) used in ERP studies, which typically presents words one at a time, is not a natural reading paradigm. Additionally, word frequency is highly correlated with word length and n-gram frequencies (such as bigram, trigram, and word-form frequencies). In some cases, the mixed use of words with frequency factors is unavoidable, and it is difficult to simultaneously control all variables in alphabetic writing systems. Evidence has demonstrated that early ERP effects are also susceptible to pre-lexical factors such as n-gram frequencies (Hauk and Pulvermüller, 2004; Hauk et al., 2006), so word frequency effects and their interactions with context on early ERP components may be attributed to form recognition of words or grams rather than actual lexical access in alphabetic language reading. By contrast, most Chinese words are comprised of two adjacent characters, which could enable us to bypass the natural confounding effects among those factors by using the advantages of Chinese two-character compounds. The study conducted by Lee et al. (2012) used two-character words as the target, but they did not explore the question of whether or not a word’s contextual predictability impacts the word processing interactively with its character frequency. The present study was designed to investigate this issue in natural Chinese reading.

It has been demonstrated that character processing is essential to, but independent from, word processing to some extent, especially for processing the end character of a two-character-word (Shen and Li, 2012), and also that the recognition of multi-character words relates to the processing of character combinations (Li et al., 2009; Zang et al., 2013; Gu and Li, 2015). Chinese words generally have no cues for their boundaries, which could pose a challenge for word segmentation during reading. The ERP technique may be disadvantageous for exploring Chinese word processing in reading due to the inability to preview upcoming words and the lack of word segmentation in RSVP reading of Chinese scripts. These limitations could be mitigated by the use of the eye movement tracking method (Sereno and Rayner, 2003; Rayner and Clifton, 2009). Research has shown that fixation time in the parent word region was also susceptible to pre-lexical variables, i.e., its character frequency (Yan et al., 2006; Li et al., 2014). Since character processing cannot be bypassed when exploring the impact of context on Chinese word processing, the question of how context impacts the earlier stages of lexical processing can be clarified, at least partly, by observing how the interaction of word predictability with the factors of character frequency impacts eye movement behaviors on the parent word region.

This study’s focus on revealing the mechanism of Chinese word processing and eye movement control during reading is valuable for at least two reasons. First, research has suggested that word segmentation during Chinese reading is a fast and early occurring process (Hoosain, 1992; Bai et al., 2008, 2013; Shen et al., 2012; Zang et al., 2013; Gu and Li, 2015). Both context and character processing have been linked to word segmentation (Yen et al., 2012; Liang et al., 2015; Zang et al., 2015; Su et al., 2016), and therefore, it is safe to speculate that interaction between contextual predictability and a word’s character frequency may be closely related to word segmentation mechanisms. Second, Chinese word recognition is assumed to involve the processing of text, words, characters, and their interactions (Li et al., 2009, 2014). Some researchers have observed processing effects (i.e., character frequency, word frequency, and predictability) separately (Rayner et al., 2005; Ma and Li, 2015; Ma et al., 2015; Zang et al., 2016; Liu et al., 2018; Wang et al., 2018a, b). Surprisingly, no study to our knowledge has orthogonally manipulated a word’s character frequency and its predictability, as we have done in the present study. The interactive view, in which context facilitates the early stages of word processing, predicts reliable interactions between word predictability and its character frequency, while the alternative modular view predicts non-reliable interactions.

Two-character words, which are the most representative Chinese words, were chosen as target words in the present study. By manipulating a word’s contextual predictability and character frequency, we checked the impact of word predictability on its processing. Two experiments were conducted, as both initial and end character processing are essential for word-form recognition despite their differences in lexical access (Li and Pollatsek, 2011; Shen and Li, 2012; Yen et al., 2012; Zang et al., 2015; Liang et al., 2015). Both experiments manipulated the variable of a target word’s contextual predictability; however, the frequency of the initial character of the target words was varied in Experiment 1, whereas the frequency of the end character was varied in Experiment 2. The effects of predictability, character frequency, and their interaction were measured by tracking readers’ eye movements. Results of eye movement metrics in the area of the target word during the first pass reading, which reflects early stages of word processing (i.e., first fixation duration, gaze, duration, skipping probability, re-fixation probability, incoming and outgoing saccade length; see Rayner, 1998, 2009; Ma and Li, 2015; Ma et al., 2015; Lin et al., 2018), were reported to check the hypotheses. Additionally, the overall pattern of interaction on word processing stages was assessed through the total reading time and regression in probability.

## Materials and Methods

### Ethical Considerations

The Center for Cognition and Brain Disorders at Hangzhou Normal University granted ethical approval to carry out the study within its facilities (Approval Number, 20190408). Participants provided written informed consent prior to their participation, and the data were anonymously collected.

### Participants

Altogether, 286 freshmen from Hangzhou Normal University participated in Experiment 1, and 282 of them participated in Experiment 2. All the participants were right-handed native Chinese speakers who had normal or corrected-to-normal vision. They were paid ¥40 for participation. None of them were aware of the purpose of the experiment or had previously participated in other similar experiments. Additionally, a group of 19 participants who did not participate in the experiments were asked to assess the predictability of the target words in the frame sentences used in the two experiments. They were given the sentence frame, not including the target word, and were asked to generate the next word in the sentence. Twenty college students from Hangzhou Normal University were asked to rate the naturalness of the sentences, and another 20 students rated the difficulty of the sentences used in both experiments.

### Apparatus

The participants’ right eye movements were recorded with an Eye Link 1000 device manufactured by SR Research Ltd, which is a form of infrared video-based tracking system that samples at a rate of 1000 Hz and has a high spatial resolution (<0.01° RMS). The sentence stimuli were presented in black on a white background. Participants sat 45 cm away from a computer screen, which was a 19-inch DELL monitor with a refresh rate of 75 Hz and 1024 × 768–pixel resolution. The sentences were displayed in Song font, with each Chinese character subtending 1.32 degrees of the visual angle.

### Procedure

Prior to beginning the experiments, participants were instructed to read the sentences to assess their comprehension and to push a button to terminate the display upon completion. Participants were randomly assigned to one of two stimulus sets and tested individually (i.e., all frame sentences in both experiments were sampled using a Latin square, thus producing two sets of stimuli). Sentences were shown to each participant in a randomized order; there was a practice block before the formal experimental session in both experiments. The aim of the practice block was to familiarize participants with the procedure before the formal experiment. Before the practice block and formal experiments, a three-point calibration of the eye-tracking system was conducted to make sure that the eye-tracker recording was accurate; in it, the participant was instructed to fixate on each of three fixation points arranged along a horizontal line across the center of the screen. Then, before reading each sentence, participants were instructed to fixate on a dot, which coincided with the position of the first character of the sentence. Concurrently, they pressed the Eye Link button (for drift correction) to start the sentence display. There were 12 practice sentences in both experiments, followed by 48 experiment sentences in Experiment 1 and another 40 experiment sentences in Experiment 2. A true/false comprehension question preceded five sentences in the practice block and 16 sentences in the formal experiments, and participants were asked to answer a Yes/No question by pressing the right or left Eye Link buttons when these questions appeared. Once the error from the drift correction of the current trial was greater than 0.5°, the eye tracker was re-calibrated before the next trial. The duration of the two experiments together was less than 40 min. Participants had no difficulty answering the questions correctly (over 90% accuracy), which indicated they were paying attention to what they were reading.

### Data Analysis

Fixation time measures on target words were analyzed first and included first fixation duration (FFD, the duration of the first fixation on the word in the first-pass reading irrespective of the number of fixations), gaze duration (GD, the sum of all first-pass reading fixation durations of a word), and total reading time (TRT, the sum of all fixation durations of a word, including first-pass and rereading time). We also analyzed fixation probability on target words, such as skipping probability (the probability of a target word not being fixated on during first-pass reading), re-fixation probability (the probability of a target word being fixated on more than once during the first-pass reading), and regression in probability (the probability of a target word being reinspected). Saccade length was also analyzed, including incoming saccade length (ISL; a progressive saccade resulting in a fixation on the target word during the first-pass reading), and outgoing saccade length (OSL; a progressive saccade launched away from the target word during the first-pass reading). It should be noted that neither the ISL nor the OSL included the cases of the re-fixation saccade.

Eye movement metrics did not have a threshold; thus, all raw data are included in the analysis. The continuous data (including FFD, GD, TRT, ISL, and OSL) were log-transformed to better fulfill the assumptions of the linear mixed-effects model (LMM). We analyzed the log-transformed data by using the LMM for continuous variables and a generalized mixed-effect model for binary variables (including skipping probability, re-fixation probability, and regression in probability) within the R environment (Baayen et al., 2008). Predictability, character frequency, and their interaction were entered as factors of fixed effect (coded as sum contrasts -1/2 vs. 1/2 for predictable vs. unpredictable and for high and low character frequency); launch site and landing positions of the first-run fixation were considered as covariates for analyzing fixation time measures. Furthermore, the launch site and their fixation durations were included as covariates when analyzing saccade length. We used maximal random effect structures, as suggested by Barr et al. (2013), with participants and stimuli as crossed random effects, but complicated models including random slopes posed a problem of convergence; therefore, the models used for analyzing continuous data were as follows: lmer (DependentVariable ∼ Predictability^∗^Frequency + covariates1 + covariates2 + (1| Participant) + (1| Item). Regression coefficients (b), standard errors (SE), t or Z (t or Z = b/SE, t for continuous dependent variables, Z for binary dependent variables), and *p*-values were reported. Models were fitted with the lme4 package (ver. 1.1-19; Bates et al., 2015), and *p*-values were estimated with the lmerTest package (ver. 3.0-1) in R (ver. 3.5.2; R Development Core Team, 2016).

### Experiment 1

#### Design and Stimuli

The experiment followed a 2 (word’s contextual predictability: predictable vs. unpredictable) × 2 (initial character frequency: high vs. low) within-subjects design. Participants read 48 sentence frames that contained the target words. Examples of the stimuli are shown in Table 1. All the target words were composed of two characters; half of the initial characters were high frequency, and the other half were low frequency. Based on a database of Modern Chinese corpus word frequency and a database of Modern Chinese corpus character frequency available from http://corpus.zhonghuayuwen.org/, word and character frequencies were calculated using occurrences per million characters as a standardized measure. The mean frequency of the initial character in the target word was more than 1000 per million characters in high character frequency conditions and less than 100 per million characters in low character frequency conditions. It was found that predictable target words were generated more than 70% of the time whereas unpredictable target words were generated less than 5% of the time; therefore, predictable target words were predicted more than unpredictable ones [*t*(47) = 27.983, *p* < 0.001].

**TABLE 1:** Example sentences in Experiment 1.

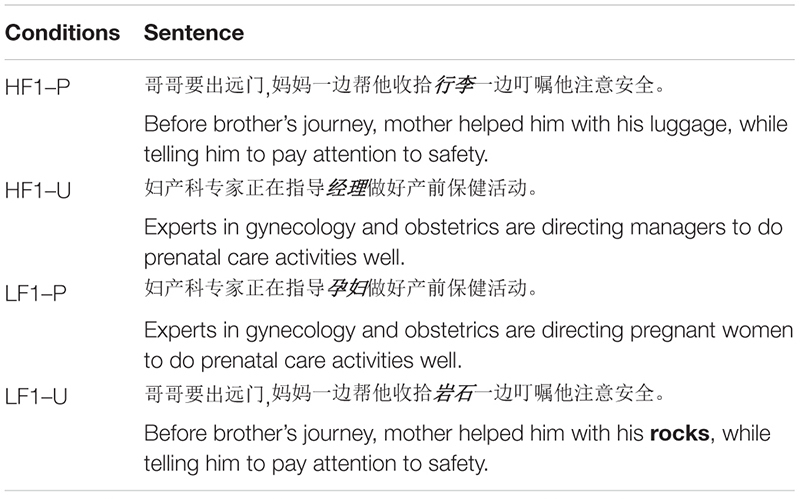

*HF1, high frequency of the first character in target word; P, predictable targets; U, unpredictable targets; LF1, low frequency of the first character in target word. Bold characters are the target words.*

Half of the target words were predictable from the prior context and half were unpredictable. As seen in Table 1, we wrote two kinds of frame sentences. The first contained a high initial character frequency with predictable target words (HF1-P) or a low initial character frequency with unpredictable target words (LF1-U). The second contained a high initial character frequency with unpredictable target words (HF1-U) or a low initial character frequency with predictable target words (LF1-P). All four target word types were balanced in terms of frequency, end character frequency, and character strokes (word frequency: *F*(3, 95) = 0.163, *p* = 0.921, end character frequency: *F*(3, 95) = 0.131, *p* = 0.941, initial character strokes: *F*(3, 95) = 0.842, *p* = 0.484, end character strokes: *F*(3, 95) = 0.516, *p* = 0.672). As seen in Table 2, there were also no differences in word predictability between HF1-P and LF1-P [*t*(46) = 0.473, *p* = 0.638] or between LF1-U and HF1-U [*t*(46) = 0.00, *p* = 1], nor were there any differences in initial character frequency between HF1-P and HF1-U [*t*(46) = 0.596, *p* = 0.554] or between LF1-U and LF1-P [*t*(46) = 1.608, *p* = 0.115]. Words before the target were also two-character words, and were balanced in terms of word frequency, character frequency, and strokes (word frequency: *t*(46) = 0.536, *p* = 0.595, initial character frequency: *t*(46) = 0.193, *p* = 0.848, end character frequency: *t*(46) = 0.16, *p* = 0.873, initial character strokes: *t*(46) = -0.219, *p* = 0.828, end character strokes: *t*(46) = -0.606, *p* = 0.547), as seen in Table 3.

**TABLE 2:** The characters of target words in Experiments 1 and 2.

Conditions	Target word in Experiment 1						Conditions	Target word in Experiment 2					
	WP	WF	FCF	SCF	FCS	SCS		WP	WF	FCF	SCF	FCS	SCS
HF1–P	76.1	12	1558	707	7.2	7.7	HF2–P	83.7	13	471	1123	7.8	8.1
	(19.2)	(12)	(1062)	(727)	(2.1)	(2.1)		(14.3)	(11)	(563)	(415)	(1.9)	(2.9)
HF1–U	0.2	10	1377	740	7.1	7.7	HF2–U	1.3	14	535	1115	7.2	7.3
	(0.11)	(11)	(1044)	(767)	(2.0)	(2.5)		(4.1)	(24)	(801)	(667)	(2.5)	(1.7)
LF1–P	78.7	10	38	641	7.0	6.9	LF2–P	83.7	15	611	47	7.1	7.8
	(19.2)	(11)	(27)	(884)	(2.3)	(2.7)		(152)	(19)	(841)	(20)	(2.5)	(2.0)
LF1–U	0.2	10	51	610	7.9	7.3	LF2–U	5	14	597	51	6.9	7.4
	(0.11)	(11)	(27)	(841)	(2.1)	(2.4)		(2.4)	(19)	(725)	(28)	(2.5)	(2.3)

*WP, Word predictable; WF, Word frequency; FCF, First character frequency; SCF, Second character frequency; FCS, First character strokes; SCS, Second character strokes. Standard deviations shown in parentheses.*

**TABLE 3:** The characters of pre-target words in Experiments 1 and 2.

Conditions	Pre-target word in Experiment 1					Conditions	Pre-target word in Experiment 2				
	WF	FCF	SCF	FCS	SCS		WF	FCF	SCF	FCS	SCS
HF1–P	53	707	857	8.1	8.1	HF2–P	22	772	911	7.8	8.4
LF1–U	(92)	(616)	(913)	(2.8)	(2.8)	LF2–U	(18)	(1218)	(1523)	(2.6)	(3.6)
LF1–P	40	675	815	8.3	8.5	LF2–P	25	600	732	8.2	8.2
HF1–U	(64)	(558)	(879)	(2.5)	(1.8)	HF2–U	(57)	(721)	(1077)	(2.7)	(2.3)

*WF, Word frequency; FCF, First character frequency; SCF, Second character frequency; FCS, First character strokes; SCS, Second character strokes. Standard deviations shown in parentheses.*

The naturalness of sentences was rated on a five-point scale, with a score of 5 indicating very natural and a score of 1 indicating very unnatural. There were no differences in the naturalness ratings among the sentence frames that contained the four kinds of target words [*F*(3, 39) = 0.03, *p* = 0.993; LF1-P: M = 4.41, *SD* = 0.99; HF1-P: M = 4.47, *SD* = 0.97; HF1-U: M = 4.40, *SD* = 0.99; LF1-U: M = 4.42, *SD* = 0.84]. Difficulty of sentences was also rated on a five-point scale, with a score of 1 indicating very easy and a score of 5 indicating very hard. There were also no differences in the difficulty ratings among the sentence frames that contained the four kinds of target words [*F*(3, 39) = 0.387, *p* = 0.763; LF1-P: M = 1.88, *SD* = 0.70; HF1-P: M = 2.04, *SD* = 0.67; HF1-U: M = 2.09, *SD* = 0.61; LF1-U: M = 2.20, *SD* = 0.72].

#### Results and Discussion

The comprehension rate for each condition was more than 90% (LF1-P: M = 94.8%, *SD* = 0.123; HF1-P: M = 96.1%, *SD* = 0.096; HF1-U: M = 96.6%, *SD* = 0.086; LF1-U: M = 95.2, *SD* = 0.112), indicating that participants fully understood the sentences and were not affected by target word predictability, initial character frequency, or their interaction (*ps* > 0.05). As shown in Tables 4, 5, reliable predictability effects were found for all of the measures: predictable targets were fixated for a shorter time, re-fixated/regressed less often, and skipped more than the unpredictable targets (FFD: predictable = 225 ms, unpredictable = 243 ms; GD: predictable = 248 ms, unpredictable = 283 ms; TRT: predictable = 281 ms, unpredictable = 404 ms; re-fixation probability: predictable = 8.0%, unpredictable = 14.0%; regression in probability: predictable = 9.9%, unpredictable = 15.9%; skipping probability: predictable = 26.8%, unpredictable = 20.8%). Saccades incoming and outgoing from predictable target words were also longer in length than those for unpredictable target words (ILS: predictable = 2.19 char, unpredictable = 2.05 char; OLS: predictable = 1.93 char, unpredictable = 1.76 char). No significant initial character frequency effects were observed for fixation time or outgoing saccade length, while significant or marginal frequency effects were observed for skip, re-fixation, and regression in the probability measures (skipping probability: high initial character frequency target word = 25.1%, low initial character frequency target word = 22.5%; re-fixation probability: high initial character frequency target word = 10.7%, low initial character frequency target word = 11.3%; regression in probability: high initial character frequency target word = 14.5%, low initial character frequency target word = 11.3%). Notably, target words with a high initial character frequency were skipped and regressed more than those with a low initial character frequency. Frequency effects were also reliable on the ISL, with longer incoming saccades observed to the target of a high initial character frequency than to a low initial character frequency (high initial character frequency target word = 2.13 char, low initial character frequency target word = 2.11 char). No reliable interaction effects were observed.

**TABLE 4:** Effects of word predictability and first character frequency on eye movement measures in Experiment 1.

Conditions	FFD (ms)	DG (ms)	TRT (ms)	Skip.Pro (%)	Refix.Pro (%)	Reg.Pro (%)	ISL (char)	OSL (char)
HF1–P	229 (38)	248 (48)	279 (116)	28.9 (17.5)	7.1 (8.8)	10.5 (10.8)	2.25 (1.00)	1.96 (0.61)
HF1–U	241 (40)	283 (70)	406 (160)	21.2 (15.7)	14.3 (13.3)	18.6 (13.8)	2.01 (0.76)	1.72 (0.59)
LF1–P	225 (43)	249 (55)	282 (114)	24.6 (15.2)	8.9 (10.5)	9.3 (9.9)	2.13 (0.87)	1.90 (0.86)
LF1–U	245 (42)	283 (68)	403 (160)	20.4 (15.3)	13.7 (13.5)	13.2 (12.1)	2.09 (0.89)	1.80 (0.57)

*Means and standard deviations (shown in parentheses) were computed across participants’ mean. Refix.Pro, re-fixation probability; Skip.Pro, skipping probability; Reg.Pro, regression in probability; ISL, incoming saccade length; OSL, outgoing saccades length.*

**TABLE 5:** Linear mixed-effects model analyses on eye movement measures of Experiment 1.

	FFD log-transformed				GD log-transformed			
	*b*	*SE*	*t*	*p*	*b*	*SE*	*t*	*p*
Intercept	2.340	0.006	372.551	<0.001***	2.413	0.008	307.919	<0.001***
F	−0.001	0.003	−0.260	0.759	0.001	0.004	0.175	0.861
P	0.029	0.003	9.599	<0.001***	0.051	0.004	14.163	<0.001***
P × F	0.020	0.014	1.510	0.138	0.009	0.019	0.447	0.657

	**TRT log-transformed**				**Skipping probability**			
	***b***	***SE***	***t***	***p***	***b***	***SE***	***t***	***p***

Intercept	2.520	0.012	203.844	<0.001***	-1.287	0.061	-21.251	<0.001***
F	-0.005	0.004	-1.183	0.273	-0.345	0.042	-8.249	<0.001***
P	0.114	0.004	26.082	<0.001***	-0.135	0.042	-3.225	0.0013**
P × F	0.013	0.036	0.313	0.755	0.181	0.178	1.015	0.310

	**Re-fixation probability**				**Regression in probability**			
	***b***	***SE***	***t***	***p***	***b***	***SE***	***t***	***p***

Intercept	-2.435	0.084	-28.831	<0.001***	2.178	0.097	-22.513	<0.001***
F	0.106	0.058	1.872	0.067^†^	-0.289	0.058	-4.993	<0.001***
P	0.679	0.058	11.742	<0.001***	0.576	0.058	9.901	<0.001***
P × F	-0.293	0.266	-1.101	0.271	-0.178	0.358	-0.496	0.62

	**ISL-log-transformed**				**OSL-log-transformed**			
	***b***	***SE***	***t***	***p***	***b***	***SE***	***t***	***p***

Intercept	0.140	0.007	18.657	<0.001***	0.279	0.018	15.771	<0.001***
F	-0.007	0.003	-2.721	0.0065**	0.005	0.004	1.281	0.200
P	-0.018	0.003	-7.168	<0.001***	-0.039	0.004	-10.534	<0.001***
P × F	0.014	0.016	0.880	0.383	0.080	0.049	1.643	0.107

F, frequency of the first character in target word; P, predictable of target word. Significant levels: ^†^p < 0.1, *p < 0.05, **p < 0.01, ***p < 0.001.

To provide further statistical support for the null interaction effect of target word predictability and initial character frequency, Bayes factor analyses for linear mixed models with fixation time and saccade length measures were conducted. Bayes factors for the full model (i.e., BFFull, the model containing the main effects of word predictability and initial character frequency and their interaction) and the model with only main effects (i.e., BFMain) were calculated. We evaluated the non-significant interaction between word predictability and initial character frequency by comparing the two models (BF = BFFull/BFMain). BF values were smaller than 1, favoring the null hypothesis; that is, word predictability had additive effects with initial character frequency. For each of the measures, we used the default scale prior (*r* = 0.5) and 10,000 Monte Carlo iterations of the Bayes Factor package (Morey et al., 2018). The results of the Bayesian analysis favored the null hypothesis. Furthermore, a sensitivity analysis with different priors (i.e., 0.2, 0.3, 0.4, 0.5, 0.6, 0.7, and 0.8) provided consistent results (all BFs < 0.67).

Pronounced predictability effects were observed on all measures of eye movements, which suggested that pervasive context predictability impacted word processing and eye movement control when reading Chinese. Measures of first fixation duration, gaze duration, skipping/re-fixation probability, and incoming saccade length were reflexes of earlier lexical processing, while regression probability and outgoing saccade length were reflexes of later processing. Thus, the predictability effects indicated a pervasive impact on the stages of word processing from pre-lexical (i.e., visual feature analyzing) to post-lexical (i.e., semantic integration) processing, or even a longer and more permanent impact when reading Chinese. No reliable frequency effects were observed for the fixation time measures, but in some saccade measures, target words with a high initial character frequency were skipped/regressed and launched longer incoming saccades than those with a low initial character frequency. It is surprising to observe that regression in probability was higher for high initial character frequency words than low initial character frequency words; we assume that this result was due to compensation for skipping the probability of the high initial character frequency word. A critical finding was that predictability and initial character frequency impacted word processing and eye movement control during Chinese reading processes additively, suggesting that no overlaps between word processing stages were impacted by word predictability or initial character frequency when reading Chinese.

### Experiment 2

#### Design and Stimuli

Like Experiment 1, Experiment 2 followed a 2 (word’s contextual predictability: predictable vs. unpredictable) × 2 (end character frequency: high vs. low) within-subjects design. Participants read 40 sentence frames that contained the target words; examples of these sentences are shown in Table 6. As in Experiment 1, predictable target words were predicted more frequently than unpredictable words [*t*(47) = 33.397, p < 0.001]. As in Experiment 1, both the target and pre-target words also comprised two characters. All four target word types were balanced in terms of word frequency, the frequency of the initial character of the target word, and character strokes [word frequency: *F*(3, 79) = 0.022, *p* = 0.996, initial character frequency: *F*(3, 79) = 0.151, *p* = 0.929, initial character strokes: *F*(3, 79) = 0.6, *p* = 0.617, end character strokes: *F*(3, 79) = 0.468, *p* = 0.705]. As seen in Table 2, there were no differences in word predictability between HF2-P and LF2-P [*t*(38) = 0.00, *p* = 1] or between LF2-U and HF2-U [*t*(38) = 0.731, *p* = 0.469]. Additionally, there were no differences in end character frequency between HF2-P and HF2-U [*t*(38) = 0.045, *p* = 0.964] or between LF2-U and LF2-P [*t*(38) = 0.461, *p* = 0.647]. Prior target words were also two-character words and balanced in terms of word frequency, character frequency, and strokes [word frequency: *t*(38) = -0.186, *p* = 0.854, initial character frequency: *t*(38) = 0.544, *p* = 0.59, end character frequency: *t*(38) = 0.43, *p* = 0.67, initial character strokes: *t*(38) = -0.417, *p* = 0.679, end character strokes: *t*(38) = 0.157, *p* = 0.876, see Table 3 for details]. Naturalness and difficulty of sentences were controlled in this experiment [analyse results for the naturalness of stimuli: *F*(3, 39) = 0.068, *p* = 0.976; LF2-P: M = 4.39, *SD* = 0.72; HF2-P: M = 4.50, *SD* = 0.45; HF2-U: M = 4.46, *SD* = 0.53; LF2-U: M = 4.48, *SD* = 0.46; analyse results for the difficulty of stimuli: *F*(3, 39) = 0.19, *p* = 0.903; LF2-P: M = 2.16, *SD* = 0.69; HF2-P: M = 2.26, *SD* = 0.69; HF2-U: M = 2.20, *SD* = 0.62; LF2-U: M = 2.04, *SD* = 0.64].

**TABLE 6:** Example sentences in Experiment 2.

Conditions	Sentence
HF2–P	演员在拍戏之前都要认真地阅读剧本以便把握剧情细节。
	Before filming, the actors must read the **script** carefully so as to grasp the details of the plot.
HF2–U	小红没有及时向房东支付现金就被赶出了房间。
	Xiao Hong was driven out of the room by landlord, because she did not pay the **cash** in time.
LF2–P	小红没有及时向房东支付房租就被赶出了房间。
	Xiao Hong was driven out of the room by landlord, because she did not pay the **rent** in time.
LF2–U	演员在拍戏之前都要认真地阅读画册以便把握剧情细节。
	Before filming, the actors must read the **cartoon** carefully so as to grasp the details of the plot.

*HF2, high frequency of the second character in target word; P, predictable targets; U, unpredictable targets; LF2, low frequency of the second character in target word. Bold characters are the target words.*

#### Results and Discussion

The mean comprehension rate for each condition was more than 90% (LF2-P: M = 95.6%, *SD* = 0.102; HF2-P: M = 94.6%, *SD* = 0.115; HF2-U: M = 94.3%, *SD* = 0.111; LF2-U: M = 94.3, *SD* = 0.119), indicating that participants fully understood the sentences and were not affected by target word predictability, end character frequency, or their interaction (*ps* > 0.05). Means and standard deviations for Experiment 2 are shown in Table 7. The results of the statistical analysis with the linear mixed-effects model are shown in Table 8. The results of the predictability effects were entirely consistent with those of Experiment 1. Significant effects of word predictability were found for all measures, with predictable targets fixated for a shorter time, re-fixated/regressed less often, and skipped more often than the unpredictable targets (FFD: predictable = 224 ms, unpredictable = 237 ms; GD: predictable = 243 ms, unpredictable = 268 ms; TRT: predictable = 271 ms, unpredictable = 396 ms; re-fixation probability: predictable = 7.1%, unpredictable = 11.0%; regression in probability: predictable = 10.1%, unpredictable = 16.2%; skipping probability: predictable = 29.1%, unpredictable = 24.9%). The length of saccades incoming and outgoing from predictable target words were also longer than those from unpredictable target words (ILS: predictable = 2.22 char, unpredictable = 2.12 char; OLS: predictable = 2.11 char, unpredictable = 2.00 char).

**TABLE 7:** Effects of word predictability and second character frequency on eye movement measures in Experiment 2.

Conditions	FFD (ms)	DG (ms)	TRT (ms)	Skip.Pro (%)	Refix.Pro (%)	Reg.Pro (%)	ISL (char)	OSL (char)
HF2–P	226 (39)	247 (54)	284 (117)	29.1 (18.9)	7.9 (11.4)	11.3 (12.3)	2.25 (1.08)	2.14 (0.72)
HF2–U	232 (42)	258 (62)	364 (168)	26.3 (16.7)	8.8 (11.3)	15.9 (13.2)	2.11 (0.94)	2.07 (0.71)
LF2–P	223 (39)	239 (50)	258 (107)	29.1 (17.8)	6.2 (9.2)	9.0 (11.4)	2.18 (0.88)	2.09 (0.79)
LF2–U	242 (49)	279 (77)	428 (198)	23.5 (17.0)	13.2 (14.4)	16.6 (14.6)	2.12 (0.90)	1.93 (0.71)

*Means and standard deviations (shown in parentheses) were computed across participants’ mean. Refix.Pro, re-fixation probability; Skip.Pro, skipping probability; Reg.Pro, regression in probability; ISL, incoming saccade length; OSL, outgoing saccades length.*

**TABLE 8:** Linear mixed-effects model analyses on eye movement measures of Experiment 2.

	FFD log-transformed				GD log-transformed			
	*b*	*SE*	*t*	*P*	*b*	*SE*	*t*	*p*
Intercept	2.334	0.007	356.361	<0.001***	2.393	0.008	289.869	<0.001***
F	0.007	0.003	2.048	0.041*	0.009	0.004	2.340	0.019*
P	0.024	0.003	6.996	<0.001***	0.036	0.004	9.341	<0.001***
P × F	0.029	0.014	2.098	0.043*	0.051	0.021	2.467	0.018*

	**TRT log-transformed**				**Skipping probability**			
	***b***	***SE***	***t***	***P***	***b***	***SE***	***t***	***p***

Intercept	2.507	0.013	186.519	<0.001***	-1.108	0.068	-16.361	<0.001***
F	0.015	0.005	2.938	0.003 **	-0.079	0.044	-1.787	0.074^†^
P	0.114	0.005	22.743	<0.001***	-0.234	0.044	-5.294	<0.001***
P × F	0.081	0.040	2.028	0.0496*	-0.149	0.221	-0.675	0.500

	**Re-fixation probability**				**Regression in probability**			
	***b***	***SE***	***t***	***P***	***b***	***SE***	***t***	***p***

Intercept	-2.735	0.010	-27.374	<0.001***	-2.391	0.117	-20.497	<0.001***
F	0.108	0.069	1.559	0.119	-0.078	0.063	-1.239	0.215
P	0.521	0.069	7.542	<0.001***	0.715	0.064	11.244	<0.001***
P × F	0.731	0.321	2.278	0.023*	0.394	0.439	0.897	0.370

	**ISL-log-transformed**				**OSL-log-transformed**			
	***b***	***SE***	***t***	***P***	***b***	***SE***	***t***	***p***

Intercept	0.130	0.009	14.449	<0.001***	0.311	0.017	18.494	<0.001***
F	0.004	0.003	1.353	0.176	-0.025	0.004	-6.121	0.008**
P	-0.017	0.003	-6.251	<0.001***	-0.023	0.004	-5.591	0.065^†^
P × F	0.049	0.025	-1.997	0.053^†^	0.023	0.044	-0.543	0.590

F, frequency of the second character in target word; P, predictable of target word. Significant levels: ^†^p < 0.1, *p < 0.05, **p < 0.01, ***p < 0.001.

The end character frequency effects were also reliable or marginally reliable for fixation times, skipping probability, and outgoing saccade length, in which readers fixated on target words with a high end character frequency for a shorter time, skipped them more often, and implemented longer outgoing saccades than for target words with a low frequency end character (FFD: words with high frequency end character = 229 ms, words with low frequency end character = 233 ms; GD: words with high frequency end character = 253 ms, words with low frequency end character = 259 ms; TRT: words with high frequency end character = 324 ms, words with low frequency end character = 343 ms; skipping probability: words with high frequency end character = 27.7%, words with low frequency end character = 26.3%; OLS: words with high frequency end character = 2.11 char, words with low frequency end character = 2.01 char). Non-significant end character frequency effects were observed for measures of incoming saccade length, regression, and re-fixation probability.

**FIGURE 1:**
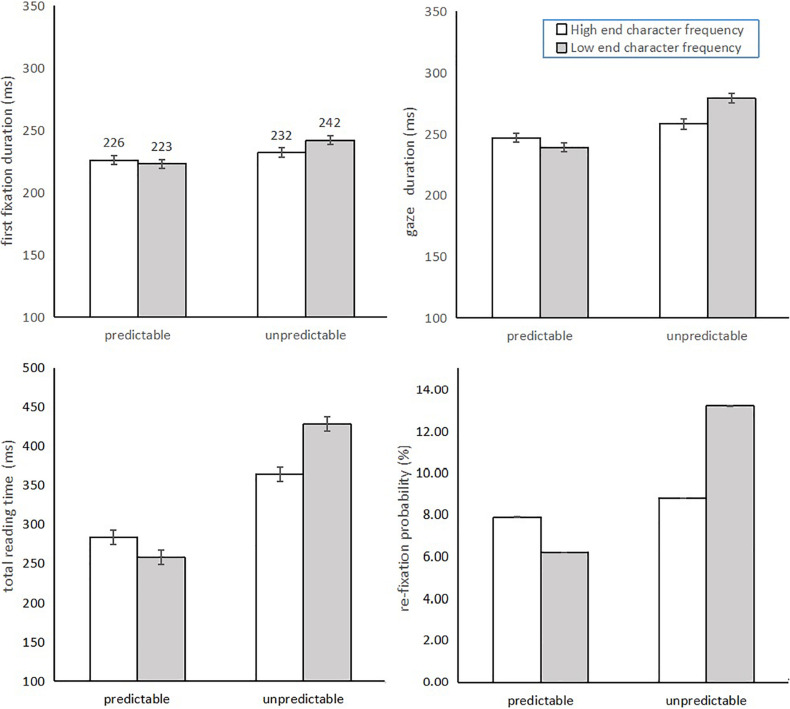
The first fixation duration, gaze duration, total reading time and refixation probability data for the four conditions.

**FIGURE 2:**
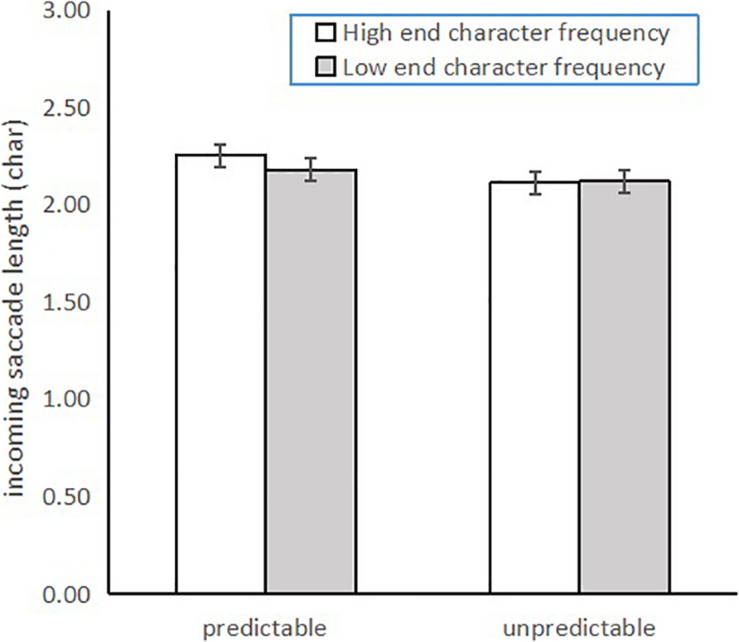
The incoming saccade length data for the four conditions.

Pervasive significant interaction effects were observed. Reliable interactions of predictability with end character frequency on fixation time and re-fixation probability were due to reliable frequency effects of the end character only when reading unpredictable target words (FFD: end character frequency effect = 10 ms, *b* = 0.022, *SE* = 0.008, *t* = 2.849, *p* = 0.006; GD: end character frequency effect = 21 ms, *b* = 0.035, *SE* = 0.011, *t* = 3.232, *p* = 0.002; TRT: end character frequency effect = 64 ms, *b* = 0.057, *SE* = 0.021, *t* = 2.758, *p* = 0.009; re-fixation probability: end character frequency effect = 4.4 %, *b* = 0.558, *SE* = 0.173, *Z* = 3.232, *p* = 0.001), but not in the reading of predictable target words (FFD: end character frequency effect = -3 ms, *b* = -0.007, *SE* = 0.008, *t* = -0.956, *p* = 0.343; GD: end character frequency effect = -8 ms, *b* = -0.016, *SE* = 0.011, *t* = -1.452, *p* = 0.153; TRT: end character frequency effect = -26 ms, *b* = -0.025, *SE* = 0.021, *t* = -1.217, *p* = 0.23; re-fixation probability: end character frequency effect = -1.7%, *b* = -0.228, *SE* = 0.172, *Z* = -1.204, *p* = 0.229). The reliable interaction of the end character frequency effect with incoming saccade length was due to a significant end character frequency effect when reading predictable target words, but not when reading unpredictable target words (end character frequency effect when reading unpredictable target word = -0.01 char, *b* = -0.049, *SE* = 0.029, *t* = -1.695, *p* = 0.098; end character frequency effect when reading predictable target word = 0.07 char, *b* = 0.066, *SE* = 0.029, *t* = 2.247, *p* = 0.03). No reliable interactions were observed for other measures. As seen from Figures 1, 2, reliable end character frequency effects on fixation time (i.e., first fixation duration, gaze duration, total reading time, and re-fixation probability) were observed only when reading unpredictable words, and an end character frequency effect on incoming saccade length was significant only when reading predictable words.

Regarding predictability effects, these results replicated the results of Experiment 1. However, the character frequency effect was slightly different from that in Experiment 1. In Experiment 2, we revealed subtle distinctions between character frequency effects, with end character frequency impacting more eye movement measures (namely, first fixation duration, gaze, duration, skipping probability, and outgoing saccade length), while initial character frequency only impacted skipping probability, re-fixation probability, regression in probability, and incoming saccade length, indicating that end character frequency effects were more pervasive than those of the initial character. No reliable impacts were evident for character frequency on refixation and regression in probability in this experiment; moreover, end character frequency was found to modulate outgoing saccade length, not the length of the incoming saccade, which is different from the saccade length results from Experiment 1. The most interesting distinctions were the reliable interaction effects in Experiment 2. Specifically, we observed the impacts of word predictability and their interactions with end character frequency on fixation measures of first fixation duration, gaze duration, total reading time, and re-fixation probability. A reliable interaction effect was also observed on incoming saccade length. In summary, the results of Experiment 2 suggest that some overlaps between word processing stages are impacted by word predictability and processing of the end characters when reading Chinese.

## General Discussion

Sufficient evidence has demonstrated that a word’s contextual predictability supplements preprocessing of a word being viewed parafoveally in alphabetic language reading (Balota et al., 1985; White et al., 2005; Schotter et al., 2012, 2015). We explored how context facilitates early stages of word processing, drawing on the advantages of two-character words in Chinese reading. Since the identification of Chinese words depends on its character processing and combinations (Li et al., 2009; Zang et al., 2013; Gu and Li, 2015), we were particularly concerned with the interaction between word predictability and its character frequency. By manipulating the predictability of target words and their character frequency, we investigated this issue in detail through two eye movement experiments. Reliable predictability effects were observed on measures of fixation time, probability, and saccade length (i.e., first fixation duration, gaze, duration, skipping probability, re-fixation probability, regression in probability, and incoming and outgoing saccade length) in both experiments, thus replicating and extending the findings of Rayner et al. (2005) and Liu et al. (2018) by suggesting a pervasive impact of predictability context on stages of word processing (i.e., from early to later stages), or even longer and more permanent impacts on Chinese reading processes. Because character processing cannot be bypassed, its frequency effects were investigated.

Frequencies of initial and end characters of two-character words were manipulated in Experiments 1 and 2, respectively. We found that end character frequency effects were more pervasive in eye movement measures than those of the initial character, contrary to results discussed previously by Yan et al. (2006), in which the impacts of initial character frequency on fixation time measures were more pronounced than those of end character frequency in two-character words. We surmised the reason for this difference was at least partly because the target words adopted in our experiments did not include low frequency words, unlike those in Yan et al. (2006). Studies have shown that the whole-word access route appears to be the dominant processing route for two-character words of medium and above frequencies, while composing two-character words from character processing only occurs when word frequency is extremely low (Liu and Peng, 1997; Shen et al., 2018). Shen and Li (2012) revealed that the processing of end characters is at least partially independent of word processing; the word superiority effect was observed only when the low-frequency initial characters in two-character words were reported. In the present experiment, all targets were of medium frequency, which may have partially eliminated initial character frequency effects because the processing of a low-frequency initial character is more dominated by the whole-word access route for words of medium frequency and above. According to Li and his collaborators, character processing is indispensable for word recognition (Li et al., 2009, 2014; Gu and Li, 2015). The distinctions between our two experiments for character frequency effects suggest different roles of the initial and end characters during word identification and extend the notion that word recognition is implemented as a process of evidence character combination (Zang et al., 2013; Ma et al., 2015).

Distinctions between character frequency effects in our experiments also extend to the issue of how character processing impacts the eye movement control in Chinese reading. Reliable or marginally reliable character frequency effects on the probability of target skipping in both experiments were observed here. A study conducted by Lin and his collaborators revealed that the decision to skip a target character can be made before integrating it into the target word (Lin et al., 2018). Additionally, it was found in the present study that skipping a two-character word was not only determined by the contextual variables of the word (e.g., its predictability) but also by the processing of its characters. Thus, these results extend the view that character skipping is based on processability, by indicating that character processing impacts the decision to skip the word to which the character belongs. Ma and Li (2015) observed that the saccade target selection was modulated by the visual complexity of the initial (not end) character of two-character words, words with low visual complexity of the initial character were skipped more often and fixation was localized nearer to the center of those words compared to words with high visual complexity of their initial characters, indicating that visual processing of the initial rather than the end character modulates the saccade toward the target word. Results of character frequency impacts on saccade length also contribute to the understanding of how character processing impacts eye movement control by clarifying that initial character frequency of a two-character word impacts saccade length toward the word, while end character frequency modulates the length of the saccade leaving the word. To summarize, our results suggest that character-processing modulates word processing effects on saccade control in Chinese reading.

The interactive hypothesis predicts reliable interactions between word predictability and its character frequency, while the modular view predicts a non-significant interaction. No reliable interaction of word predictability with its initial character frequency was observed in Experiment 1, while pronounced interaction effects of predictability with end character frequency were observed in Experiment 2 on measures of fixation time, re-fixation probability, and incoming saccade length. It is unacceptable to conclude that word predictability has no impact on character processing, because a large body of evidence supports the impact of word predictability on the early stages of parafoveal processing (Balota et al., 1985; White et al., 2005; Schotter et al., 2012, 2015; Liu et al., 2018). Considering that initial character processing was seriously affected by the word superiority effect (Shen and Li, 2012), the impact of initial character frequency and its interaction with word predictability may be subsumed in the effects of word factors, such as word frequency, concreteness, and acquired age, especially in tasks when participants have enough time to process the word (i.e., during reading). The results of Experiment 2 suggest an interactive explanation (Morton, 1969; McClelland, 1987), in which context directly impacts character processing when reading Chinese. Thus, the results extend the view that predictability facilitates the visual early stages of word processing (Lee et al., 2012), as well as the shift to character processing (i.e., its form perception or recognition) stages.

It will be valuable to explore why end character frequency effects were more susceptible to modulations from predictability than those of initial character frequency. The interaction differences between the two experiments may enrich the understanding of the mechanisms underlying word segmentation for several reasons. First, the early/parafoveal occurrence of word segmentation was revealed (Gu and Li, 2015; Su et al., 2016); among the processes underlying word predictability, end character frequency effects, and word segmentation may overlap in the time window. Second, end character processing was more closely related to word segmentation than initial character processing. For instance, as mentioned before, inserting a space after the end character of a target word facilitates its processing, whereas inserting a space before the initial character does not have such a facilitating effect (Liu and Li, 2012). Instead, statistical cues, such as the probability of a character’s being in the end position in a word, are mainly used for word segmentation (Yen et al., 2012; Liang et al., 2015; Zang et al., 2015). Third, it was proposed that contextual information is used for Chinese word segmentation during reading (Li et al., 2009). Incoming saccade length is an index of parafoveal word processing/segmentation. We observed that predictability enhanced end character frequency effects on incoming saccade length. Thus, it is reasonable to conclude that the interaction of word predictability with end character frequency may be one of the mechanisms for word segmentation in Chinese reading. Of course, further studies are still needed to confirm this speculation.

Increasing evidence suggests an additive impact pattern of word predictability and its frequency in reading alphabetic texts (Rayner et al., 2004, 2006; Miellet et al., 2007; Gollan et al., 2011; Slattery et al., 2012), while a few studies observed an interactive impact pattern, especially among participants with lower reading proficiency levels (Ashby et al., 2005; Hand et al., 2010). Compared to alphabetic texts, Chinese texts are more information-dense. Investigations have revealed that more processing mechanisms, such as word segmentation and character processing (Bai et al., 2008; Li et al., 2009, 2014; Shen et al., 2012; Zang et al., 2013), are needed for accessibility of multi-character words in Chinese than for words in alphabetic languages. Furthermore, an overlapping perceptual span during reading processes was also observed, which is not the case for alphabetic languages (Inhoff and Liu, 1998), implying that Chinese scripts may be more difficult to encode from bottom-up processing than alphabetic text. Therefore, normal adult Chinese readers may be more dependent on context for word identification, allowing a more convenient evaluation of interaction patterns of predictability and frequency factors. This research enhances our understanding of interactive compensatory processing theories suggesting that the human cognition system can compensate for the inefficiencies of bottom-up processing by posing more demands on other information sources, such as sentence context (Stanovich, 1986), thus highlighting the need for a more interactive reading strategy for Chinese reading processes.

Several limitations of the present study must be acknowledged. First, we constructed two kinds of framed sentences: the HF-P and the LF-U conditions had different sentence prefixes than the LF-P and HF-U conditions, which may have given rise to differential spill-over effects that introduced spurious interaction effects or masked true interaction effects. We controlled these differential spill-over effects as much as possible – prefix words of the framed sentences word were controlled to two characters in length and were also balanced in terms of word frequency, character frequency, and strokes. Thus, we eliminated their interactions with the target word as much as possible. Second, we did not take care to avoid the situation in which a lexical associated word/character appeared in the context preceding the target; however, 19 college students were asked to assess the predictability of all the target words in the frame sentences and found no differences in word predictability between HF-P and LF-P or between LF-U and HF-U in either experiment. Third, we tested our hypothesis in two experiments but only observed reliable interactions in Experiment 2. The lack of an interaction of word predictability with initial character frequency is theoretically acceptable. Additionally, a large sample was used in the study, and the interactions were tested multiple times (eight eye movement measures were used) in Experiment 2. Although the extent to which statistical power was reduced is unclear, five reliable interactions from the eight measures are sufficient to confirm that prediction of a word facilitates the processing of its characters in Chinese natural reading.

In summary, our study explored the nature of the interaction between word predictability and the frequency of its compound characters in Chinese reading. The results provide worthwhile approaches for validating models of word processing and eye movement control when reading Chinese. As the most prominent models, both E-Z Reader and SWIFT are used in modeling word processing and eye movement control in alphabetic languages during the reading process. Regarding the interaction between word frequency and predictability, the E-Z Reader model changed its multiplicative function to an additive one (Pollatsek et al., 2006), whereas a word frequency–predictability multiplicative interaction would be expected within the SWIFT model, since it identified a different temporal profile for the functions of word predictability and frequency (Engbert et al., 2005; Hand et al., 2010). Both models, however, are deficient in modeling the word processing and eye movement control of Chinese readers due to the lack of modules on character processing and word segmentation. A specialized model for Chinese reading, proposed by Li and his colleagues (Li et al., 2009), may have a greater ability to explain the present data, by implementing multiple levels of the process (i.e., a visual feature level, character level, word level) and assuming interactive relations between any two adjacent levels. However, this leaves open the issue of how context interacts with bottom-up processing. In summary, the results of the present study imply that contextual effects and their interaction with bottom-up processing (character processing) are needed to account for text processing and eye movement behavior in Chinese reading.

## Data Availability Statement

The datasets for this article are not publicly available because the authors intend to use the data in future research. Requests to access the datasets should be directed to ZL, lzhf2008@163.com.

## Ethics Statement

The studies involving human participants were reviewed and approved by the Center for Cognition and Brain Disorders, Hangzhou Normal University. The patients/participants provided their written informed consent to participate in this study.

## Author Contributions

ZL conceived and designed the experiments, performed the experiments, analyzed the data, wrote the manuscript, and prepared the tables. XL wrote the manuscript and reviewed drafts of the manuscript. WT contributed reagents, materials, analysis tools, and reviewed drafts of the manuscript. FF made sentences stimuli and performed the experiments. All authors contributed to the article and approved the submitted version.

## Conflict of Interest

The authors declare that the research was conducted in the absence of any commercial or financial relationships that could be construed as a potential conflict of interest.
